# Pilot-scale fermentation to produce trichodiene, a fungal volatile that suppresses trichothecene production in mycotoxigenic fungi

**DOI:** 10.1128/aem.01695-25

**Published:** 2026-01-15

**Authors:** William T. Hay, Nathan D. Kemp, Angela R. Payne, Nicholas Rhoades, Guixia Hao, Martha M. Vaughan, Susan P. McCormick

**Affiliations:** 1 USDA, Agricultural Research Service, National Center for Agricultural Utilization Research, Mycotoxin Prevention and Applied Microbiology Research Unit, Peoria, Illinois, USA; 2 USDA, Agricultural Research Service, National Center for Agricultural Utilization Research, Crop Bioprotection Research Unit, Peoria, Illinois, USA; Michigan State University, East Lansing, Michigan, USA

**Keywords:** trichodiene, *Fusarium sporotrichioides*, fermentation, biofumigation, confocal microscopy, xanthotoxin, deoxynivalenol, pilot scale

## Abstract

Trichodiene is a sesquiterpene hydrocarbon and the precursor of trichothecene mycotoxins produced by *Fusarium* and other fungi. Interestingly, utilizing trichodiene as a volatile treatment has been shown to reduce mycotoxin production in *Fusarium graminearum*-infected wheat heads. This research developed a pilot-scale fungal fermentation method to produce trichodiene as a biofumigant to mitigate mycotoxin contamination. A *TRI4* mutant strain of *Fusarium sporotrichioides* was used to mass-produce trichodiene. Xanthotoxin, a furanocoumarin produced by parsnips, was used to enhance trichodiene production in this mutant. Xanthotoxin treatments significantly increased trichodiene yield and were found to promote lipid droplet release from the fungal germlings. Benchtop scale evaluations were conducted to determine the impacts of xanthotoxin concentration, fermentation time, extraction solvent, and filtration on overall yield. After optimal conditions were identified, the fungal cultures treated with xanthotoxin were fermented for 1 week in a 30 L bioreactor. After an organic extraction of the fungal culture and concentration of the extract, trichodiene was isolated using silica gel column chromatography. Purified trichodiene reduced mycotoxin production in *F. graminearum* in a dose-dependent manner. This research will allow the production of trichodiene in sufficient quantities to further evaluate its efficacy as a biofumigant to suppress mycotoxin production in *F. graminearum*.

**IMPORTANCE:**

Food contamination from microbial toxins is a threat to human and animal health. Globally, the pathogen *Fusarium graminearum* causes annual losses in billions of dollars for cereal farmers and producers. Previous studies have shown that the fungal terpene trichodiene can suppress the production of vomitoxin (deoxynivalenol) by *F. graminearum*. We developed a way to scale production of trichodiene and use it to inhibit *F. graminearum* toxin contamination. A mutant strain of *Fusarium sporotrichioides* that produces trichodiene was grown in large 30 L fermenters and treated with xanthotoxin, a natural compound made by parsnips. Xanthotoxin caused lipid droplet release from the fungus and increased trichodiene yield. The purified trichodiene effectively reduced toxin production by *F. graminearum* by direct contact or as a volatile. Based on these research findings, trichodiene can be produced using common large-scale fermentation methods. Field formulations can now be developed to suppress mycotoxin contamination in food and feed.

## INTRODUCTION

Fungi produce a multitude of natural products, some of which are important for material, food, and pharmaceutical sciences (1, 2). However, fungal pathogens can produce mycotoxins, harmful natural products that are a significant health hazard for animal and human consumers (3, 4). Globally, mycotoxins cause significant annual agricultural losses and harm human health and development (5–7). To improve food safety and security, it is imperative to identify helpful natural products and methods to produce them to control mycotoxin contamination of crops.

Trichothecenes are sesquiterpenoid mycotoxins primarily produced by *Fusarium* plant pathogens that infect common cereals such as wheat, barley, rye, oats, and corn (8, 9). During infection, the grain can become contaminated with mycotoxins—commonly deoxynivalenol (DON)—making them unsafe for food or feed. Consumption of contaminated grain can lead to vomiting, gastrointestinal inflammation, immune suppression, and an increased risk of bacterial enteritis (10–12).

It was recently reported that trichodiene, the volatile hydrocarbon precursor of trichothecenes (13), inhibited the biosynthesis of the trichothecene DON in *Fusarium graminearum* (14). In that study, trichodiene was released as a volatile by a *Trichoderma harzianum* strain which had been engineered to express the *Fusarium* terpene cyclase gene *TRI5*. Trichodiene is not phytotoxic and is not associated with pathogenicity (15). During trichothecene biosynthesis, trichodiene is initially oxygenated using a cytochrome P450 monooxygenase encoded by *TRI4* (16). The P450 adds hydroxyl groups to trichodiene at the C2, C3, and C11, and an epoxide at C12,13. This epoxide is characteristic of all trichothecenes and is essential for their cytotoxicity (17). The trichothecene epoxide moiety has a high affinity for the active site of eukaryotic ribosomes, blocking protein synthesis and ultimately causing cell apoptosis (18). However, without a functional Tri4, trichodiene accumulates within the fungus (19, 20). Currently, trichodiene is not available for purchase from any chemical supplier. Trichodiene can be synthesized but requires a complex multistep process and careful control of experimental conditions to produce a limited yield of a non-racemic mixture (like that produced naturally) (21–24). Establishing pilot-scale production of promising fungal compounds typically requires numerous complex process developments. Therefore, our goal was to develop an efficient method to produce and isolate trichodiene at a pilot scale for use as a biofumigant to control mycotoxin contamination by *Fusarium* pathogens.

To mass-produce trichodiene, a *TRI4* knockout *Fusarium sporotrichioides* mutant (F15) was selected. *TRI4* encodes a multifunctional oxygenase, and the *TRI4* mutant strain is unable to convert trichodiene into the oxygenated T-2 toxin biosynthetic intermediate isotrichotriol, thus accumulating trichodiene instead of the dangerous T-2 toxin (20). To increase overall trichodiene production, xanthotoxin, a furanocoumarin phytotoxin from parsnip (*Pastinaca sativa*), was added to the growth media. Xanthotoxin has been previously demonstrated to block P450 oxygenations in *Fusarium* trichothecene biosynthesis, causing accumulation of trichodiene (25, 26). Interestingly, an increase in trichodiene production with xanthotoxin was also reported in *Fusarium TRI4* mutant strains that accumulate trichodiene (20). In the present study, different trichodiene isolation methods, including physical filtrations and varying organic solvent extractions, were evaluated to optimize yield. Finally, we evaluated the efficacy of the purified trichodiene as a contact and biofumigant treatment to reduce mycotoxin biosynthesis in the fungal pathogen *F. graminearum*.

## MATERIALS AND METHODS

### Fungal strains

The strains used in this study include *F. sporotrichioides* NRRL 3299 (cross-reference ATCC 24631, FRC T-423, FRC T-424, MRC 1768, and MRC 43) and *F. sporotrichioides* F15, a *TRI4* disruption mutant of NRRL 3299 that accumulates trichodiene (19). Strain F15 was used in all trichodiene production procedures, while NRRL 3299 acted as a control comparison during confocal microscopy imaging. *F. graminearum* NRRL 22917 (cross-reference GZ3639; R. L. Bowden, Kansas State University) (27) was used to evaluate the efficacy of trichodiene to inhibit trichothecene mycotoxin production. F15 was used to produce isotopically labeled trichodiene. Finally, *F. graminearum* mutant strain B4-1 (28) was used to produce isotopically labeled 15-acetyldeoxynivalenol (15ADON) and for contact and fumigation assays with labeled trichodiene.

### Fungal culturing and spore inoculum production

*F. sporotrichioides* species were removed from long-term storage conditions (−80°C, 50% glycerol), transferred to a V8 agar plate (2% agar, 20% V8 juice, 0.3% CaCO_3_) and stored at 25°C, 12 h/day of fluorescent light (60 µmol/m^2^/s^1^) supplemented with UV-A. Following a 10-day incubation, 1 cm^2^ agar pieces were used as inoculum for flask cultures or as the inoculum for spore production.

For *F. sporotrichioides* spore inoculum production, two 1 cm^2^ agar chunks were transferred to mung bean broth (MBB, 4% mung bean) and stored for 3 days at 28°C, 200 revolutions per minute (rpm), under dark conditions. The 3-day-old cultures were filtered of solids <40 µm, centrifuged at 4,200 rpm for 10 min, decanted, resuspended in sterile deionized water (SDI) for rinsing, centrifuged and decanted again, resuspended in SDI, counted for cell density on a hemocytometer, and adjusted to a final spore concentration of 1 × 10^5^ or 1 × 10^6^ spores/mL in SDI. *F. graminearum* spore inoculum was produced in the same manner using 3-day growth on MBB and adjusted to a final spore concentration of 1 × 10^5^ spores/mL in SDI.

### Small-scale fermentation

Small-scale benchtop fermentations were used to evaluate the effect of xanthotoxin concentrations on trichodiene production by the *F. sporotrichioides* mutant strain F15. F15 was grown in 5GYEP liquid media (5% glucose, 0.1% yeast extract, 0.1% peptone; 100 mL in 250 mL Erlenmeyer flask), after inoculation with two 1 cm^2^ agar chunks. Flasks were incubated for 4 days at 28°C, 200 rpm, in the dark. After 24 h, xanthotoxin (8-methoxypsoralen, Sigma) dissolved in acetone was added to cultures for final concentrations of 0, 0.05, 0.1, 0.2, and 0.3 mM; each concentration was evaluated in triplicate. On days 4, 5, 6, 7, and 14 post-inoculation, cultures were removed from incubation, shaken to evenly suspend fungal growth in the media, then 5 mL aliquots were removed from the flasks. To assess solvent extraction efficiency, day 4 aliquots were mixed with 2 mL of either ethyl acetate or hexane, vortexed for 30 s, and centrifuged at 4,200 rpm for 10 min. Then, 1 mL of extract was transferred into a 2 mL, 9 mm glass autosampler vial and analyzed for trichodiene content using gas chromatography-mass spectrometry (GC-MS). Ethyl acetate was used as the sole extraction solvent for the remaining sample time points.

We also evaluated whether filtering the culture could minimize the amount of solvent needed for the extraction of trichodiene. Small-scale benchtop fermentations of *F. sporotrichioides* in 5GYEP and 0.2 mmol/L xanthotoxin were grown in triplicate following the procedure described previously. After 7 days, the cultures were filtered through a 70 mm Whatman #54 filter in a Buchner funnel. The mycelial masses were weighed and then extracted in ethyl acetate as stated above. The amounts of trichodiene in mycelial masses were compared to amounts in filtered media and the whole culture.

### GC-MS analysis identification and quantification of trichodiene

Fungal extracts were analyzed directly without derivatization by GC-MS with an Agilent 8860 gas chromatograph fitted with an HP-5 MS column (30 m, 0.25 mm, 0.25 μm), a 5977 mass detector, and MassHunter Workstation v.10 software. The carrier gas was helium, and the samples were injected with a 20:1 split ratio. The oven was held at 150°C for 1 min after injection, heated to 280°C at 30°C/min, and then held at 280°C for 3.7 min. Under these conditions, trichodiene and 15ADON eluted at 3.3 and 6.8 min, respectively. Trichodiene and 15ADON were quantified using standard curves prepared from fungal cultures as described previously (15).

### Confocal microscopy

The small-scale fermentation procedure was repeated as above using *F. sporotrichioides* F15 grown with xanthotoxin (0, 0.05, 0.1, 0.2, or 0.3 mM) added after 1 day. As controls, *F. sporotrichioides* NRRL 3299 was grown as described above with 0 or 0.2 mM xanthotoxin.

At days 4, 7, 8, 9, and 10, 5 mL aliquots of each culture were transferred to 15 mL polystyrene conical tubes. A 1 mg/mL stock solution of Nile Red (Sigma, CAS 7385-67-3) dissolved in acetone was used to make a working solution of 0.5 µg/mL Nile Red in phosphate-buffered saline. A 25 µL subsample from each treatment was stained in 500 µL of the working solution for 10 min in dark conditions at room temperature. Using a 50% glycerol (Sigma-Aldrich, CAS 56-81-5) mounting solution, 25 µL of stained culture was mounted onto 25.0 × 75.0 × 1.0 mm glass slides (Fisher Scientific) covered with 18 × 18 mm no. 1 glass cover slips (VWR International LLC). Samples were imaged on a Zeiss Axio Observer 7 fitted with a Zeiss LSM 900 with Airyscan 2 and Zeiss LSM T-PMT using ZEN Blue Edition 3.5 software for image capture, analysis, and processing.

### Pilot-scale fermentation

**Fig 1:**
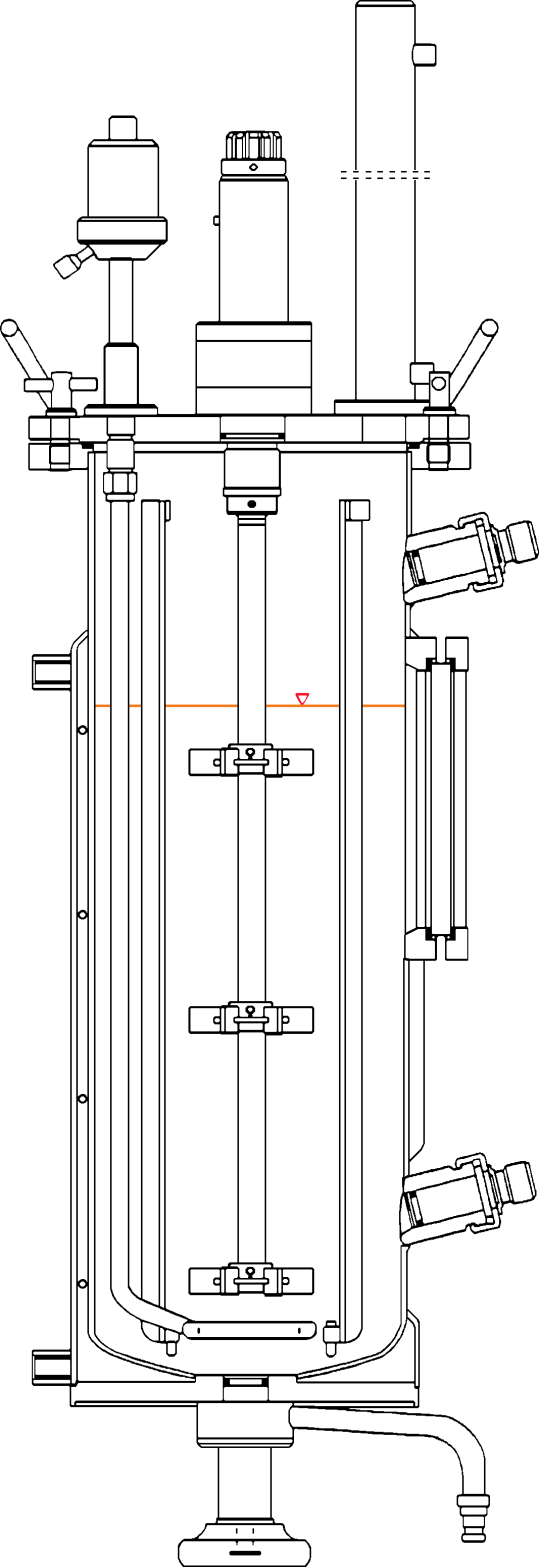
Fermenter diagram (C30-2), 30 L working volume (as indicated by the red triangle), total volume 42 L.

Pilot-scale fermentations were conducted in triplicate using a 30 L bioreactor (C30-2; B. Braun, Allentown, PA, USA; Fig. 1). The 5GYEP growth media were sterilized inside the baffled drum. The inoculum (30 mL of 1 × 10^6^ spores/mL F15 spore inoculum in SDI) was added to the sterile media via a cannula through a top port septum for a final concentration of 1 × 10^3^ spores/mL. Three Rushton impellers were used for agitation. The parameters for the bioreactor were temperature (28°C), agitation (200 rpm), and aeration (5 standard liters per minute [slpm]). The pH was uncontrolled during the growth of the *F. sporotrichioides* culture. To be able to monitor fungal production progress, bioreactor run data for pH, pO, rpm, air (slpm), and temperature (°C) was collected. After 24 h of growth, 30 mL of xanthotoxin dissolved in acetone was added to the bioreactor using the same method as the inoculum, bringing the final fermentation concentration of xanthotoxin to 0.2 mmol/L. Samples were taken from the sterile sample port at the bottom of the bioreactor on days 3, 4, 5, 6, and 7 post-inoculation. Samples were extracted in ethyl acetate for trichodiene analysis using GC-MS and filtered for fungal biomass accumulation. After 7 days of fermentation, the culture was removed from the bioreactor and stored at 4°C until processing.

### Manual solvent extraction and isolation of trichodiene

The fermentation product was extracted in a 2:1 ratio of culture to ethyl acetate. Once combined in a glass separatory funnel, the mixture was vigorously agitated for 1 min, then allowed to settle for 15 min. The top ethyl acetate layer was separated from the fungal culture media and filtered to remove any remaining debris. The extract was concentrated with rotary evaporation.

The concentrated extract was resuspended in hexane, and trichodiene was purified over a silica gel column (high purity grade, pore size 60 A, 70–230 mesh; Sigma-Aldrich, CAS 112926-00-8) eluted with hexane. Fractions were monitored for trichodiene with GC-MS. Trichodiene was purified to >95% as determined by GC-MS analysis.

### Trichodiene fumigation and contact assays vs *F. graminearum*

*In vitro* contact and fumigation assays were carried out to assess the ability of trichodiene to inhibit *F. graminearum* growth and trichothecene production on media. Agmatine agar medium was chosen for *F. graminearum* growth as a strong inducer of trichothecene biosynthesis (29). This medium contained sucrose and agmatine sulfate as the sole nitrogen source. For the contact assays, 0.03, 0.3, 3.0, and 30.0 mg of purified trichodiene, diluted in acetone, was directly mixed into 10 mL agmatine agar media and poured into 60 × 15 mm polystyrene petri dishes for desired experimental concentrations (0.003, 0.03, 0.3, and 3.0 mg/mL). A non-treatment control was also prepared by mixing an equal volume of acetone solvent into the media (0 mg/mL of trichodiene). The trichodiene and control agmatine agar plates were inoculated in the direct center with 10 µL of *F. graminearum* spore inoculum (1 × 10^5^ spores/mL *F. graminearum* Gz3639). The *F. graminearum* spore inoculum was prepared following the protocols described in “Fungal culturing and spore inoculum production,” above.

Polystyrene I-plate petri dishes (89137-760; VWR International, Radnor, PA, USA), which contained a center barrier, separating the plate into two equal halves, but allowed volatile exchange between the two sides through a gap at the top, were used for the fumigation assays. To each I-plate, 10 mL of agmatine agar media was added to one side and inoculated with 10 µL of *F. graminearum* spore inoculum (1 × 10^5^ spores/mL). On the empty side, trichodiene diluted in acetone was added to achieve the desired concentrations (0.6, 6.0, 60.0, and 600.0 µg/cm^3^). The container airspace was approximately 50 mL. A non-treatment control (0 µg/cm^3^ trichodiene) was also prepared using the equal volume of acetone. All assays were replicated 5 times, with the highest dose treatments replicated 10 times.

Both the contact and fumigation assay plates were sealed with Parafilm and incubated for 7 days at 25°C in the dark. After 7 days, images of each plate were taken with a Nikon DCMR D5600 equipped with an AF-P Nikkor 18-55 mm 1.0:3.5–5.6 G lens, set 40 cm above the base of the sample. Fungal growth area was measured using Image J software (National Institutes of Health, Bethesda, MD, USA). Whole-sample media and fungal growth were extracted and analyzed for mycotoxin content using GC-MS.

To evaluate whether trichodiene fumigation would be harmful to plants, spring wheat seeds (variety Norm) were exposed to trichodiene as a fumigant, modified from previously described protocols (30). Briefly, the wheat seeds were surface sterilized with 1:10 bleach/sterile water for 6 min and triple washed with sterile water, and excess water was removed using a Buchner funnel. To one side of the I-plate, 10 mL of water agar media was added. After cooling, a set of 20 wheat seeds was placed on the water agar. On the other side of the I-plate, trichodiene diluted in acetone was applied to a Whatman #1 filter paper (approximately 1 cm^2^). Trichodiene treatment concentrations were 0, 0.4, 4.0, 40.0, and 400.0 µg/cm^3^. The plates were sealed with Parafilm and stored in the dark for 7 days at 25°C (*n* = 5). After 1 week, germinated wheat seeds were counted.

### Trichothecene extraction and GC-MS determination

After 7 days of growth, agmatine cultures were cut into 1 cm^2^ pieces and transferred to 50 mL polystyrene conical tubes. Samples were extracted with 6 mL ethyl acetate shaking on a vertical vortex for 5 min at 1,700 rpm and then centrifuged at 3,000 rpm for 5 min. The top organic layer was transferred to a 1-dram glass vial to dry down under air using a 55°C heat block. Dried samples were resuspended in 1 mL ethyl acetate and transferred to a 2 mL, 9 mm glass vial for GC-MS. Purified trichothecene was used to generate total ion chromatograms for toxin quantitation (31).

### C^13^-labeled trichodiene

To determine whether the volatile trichodiene could be incorporated into the trichothecene biosynthesis pathway, ^13^C-labeled trichodiene was produced via fermentation and used to fumigate *F. graminearum*. ^13^C_15_-trichodiene was isolated from liquid cultures of the *F. sporotrichioides TRI4* mutant strain, F15, grown on ^13^C-YEPD medium prepared with ^13^C glucose (50g ^13^C_6_ glucose from Cambridge, 1 g peptone, 1 g yeast extract/L of water). Liquid cultures were grown for 7 days at 28°C with shaking at 200 rpm. Cultures were extracted with ethyl acetate, and the concentrated extract was purified on a silica gel column eluted with hexane. Column fractions were monitored with GC-MS. The mass spectrum of trichodiene has a molecular ion of *m*/*z* 204 and a characteristic fragment ion at *m*/*z* 109; the ^13^C_15_-trichodiene has a molecular ion at *m*/z 219 and a characteristic fragment ion at *m*/*z* 117. ^13^C_17_-15ADON was isolated from liquid cultures of *F. graminearum* mutant strain B4-1 (28) grown on ^13^C-YEPD medium prepared with ^13^C_6_ glucose. Cultures were grown for 7 days at 28°C with shaking at 200 rpm. Cultures were then extracted with ethyl acetate, and the concentrated extract was purified on a silica gel column eluted with dichloromethane:methanol (98:2). Column fractions were monitored with GC-MS. The mass spectrum of 15ADON has a molecular ion at *m*/*z* 338 and characteristic fragment ions at *m*/*z* 249, 231, 203, and 79. The ^13^C_17_-15ADON isolated from cultures grown on ^13^C-YEPD had a molecular ion of *m*/*z* 355 and characteristic fragment ions at *m*/*z* 263, 245, 216, and 85. Cultures grown on YEPD medium were fumigated with ^13^C_15_-trichodiene, and cultures grown on ^13^C-YEPD medium were fumigated with ^12^C_15_-trichodiene. Fumigated cultures were extracted following the previously described protocols and analyzed via GC-MS to determine whether externally applied trichodiene was incorporated into trichothecene biosynthesis.

### Statistical analysis

The impact of xanthotoxin concentration on trichodiene production and fungal biomass was evaluated using Dunnet’s test (*α* = 0.05, JMP v.17.0). Differences in fungal area, fungal biomass, and 15ADON accumulation were evaluated using Student’s *t*-test (*α* = 0.05, JMP v.17.0) to determine significant differences between treatments. Linear correlations between independent variables were fit and evaluated using JMP v.17.0. Information on pairwise comparisons can be found within the table and figure legends.

## RESULTS

### Impact of xanthotoxin on fungal biomass and trichodiene production

In this study, the *F. sporotrichioides* mutant strain F15 produced trichodiene in liquid culture and produced significantly more trichodiene when cultured with xanthotoxin. Fungal fermentations are often started with a media-plug inoculation of the desired strain, but this can cause significant variability in inoculum concentration and difficulties in upscaling the process. Therefore, we evaluated the use of *F. sporotrichioides* spores harvested from mung bean broth, diluted to 1 × 10^5^ spores/mL, as an inoculum for our fermentations. We found that the spore inoculum produced approximately 70% greater biomass yields than agar plug inoculations (Table S1). Spore inoculations were used for all subsequent fungal fermentations.

**Fig 2:**
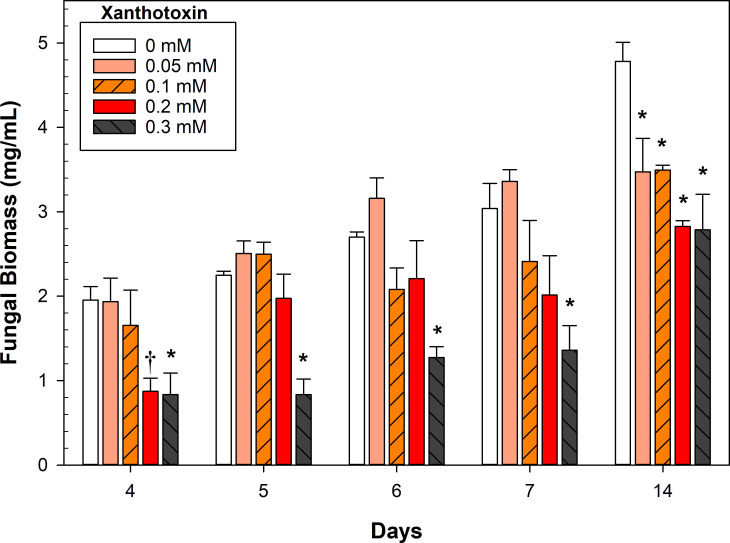
Impact of xanthotoxin concentration on fungal biomass accumulation. Error bars represent SE. The symbols † and * indicate significant differences with the control treatment at a given day (0 mM) at *α* = 0.1 and 0.05, respectively, as determined by Dunnett’s test (*n* = 3).

Benchtop shaker fermentations were evaluated to determine the effect and ideal concentration of xanthotoxin to maximize the yield of trichodiene. Xanthotoxin enhanced the production of trichodiene but caused decreased fungal biomass accumulation (Fig. 2) in a concentration-dependent manner (*r*^2^ = 0.58, *P* = 0.0009 at day 7). The highest xanthotoxin concentration tested, 0.3 mM, decreased fungal biomass by approximately 50%, compared to the control.

We extracted trichodiene with hexane and ethyl acetate and evaluated their extraction efficiencies. Ethyl acetate was a more effective extraction solvent than hexane, yielding approximately twice as much trichodiene in nearly all samples (Fig. S1). We also evaluated whether we could minimize the solvent needed—extracting only the mycelia—by first physically separating the fungal hyphal mass from the culture via filtration. Unfortunately, significant amounts of trichodiene were found in the culture media after filtration, with yields reduced by an average of 72%, compared to the whole culture extractions with ethyl acetate. Therefore, for all subsequent culture extractions, we used ethyl acetate on the unfiltered bulk media.

**Fig 3:**
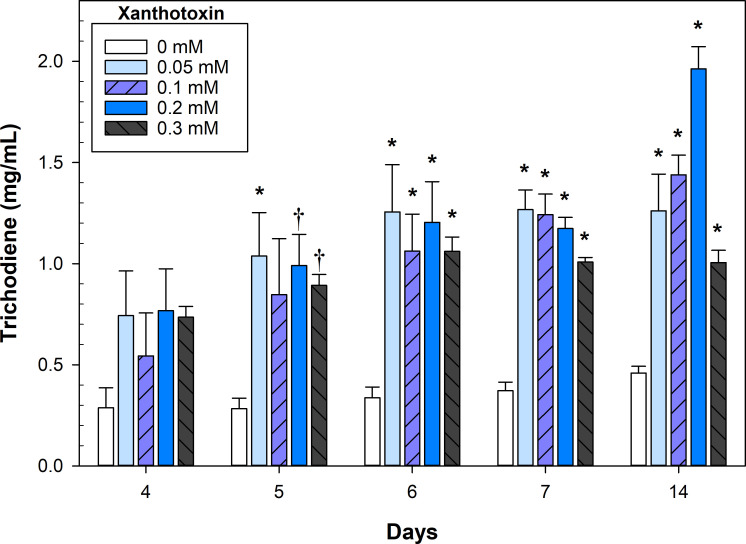
Trichodiene yield from *F. sporotrichioides* grown with varying concentrations of xanthotoxin. Error bars represent SE. The symbols † and * indicate significant differences with the control treatment at a given day (0 mM) at *α* = 0.1 and 0.05, respectively, as determined by Dunnett’s test (*n* = 3).

Without the addition of xanthotoxin, trichodiene yields were poor and remained low despite significant biomass accumulation over time (0 mM xanthotoxin, Fig. 3). Overall, the addition of any concentration of xanthotoxin increased trichodiene yields by approximately threefold. Most fungal cultures reached peak yields by day 6 or 7. The 0.1 and 0.2 mM xanthotoxin treatment had increased trichodiene accumulation at 2 weeks, but the increase from days 7 to 14 was only statistically significant in the 0.2 mM culture (*P* = 0.003). The 0.3 mM xanthotoxin culture doubled its biomass from days 7 to 14 (Fig. 2), but trichodiene yields remained constant (Fig. 3).

**Fig 4:**
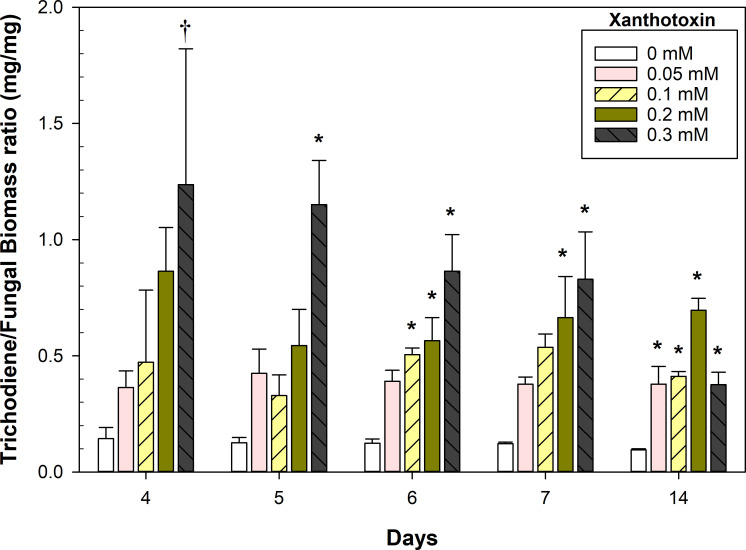
Trichodiene yield to fungal biomass ratio. Error bars represent SE. The symbols † and * indicate significant differences with the control treatment at a given day (0 mM), *α* = 0.1 and 0.05, respectively, as determined by Dunnett’s test (*n* = 3).

**Fig 5:**
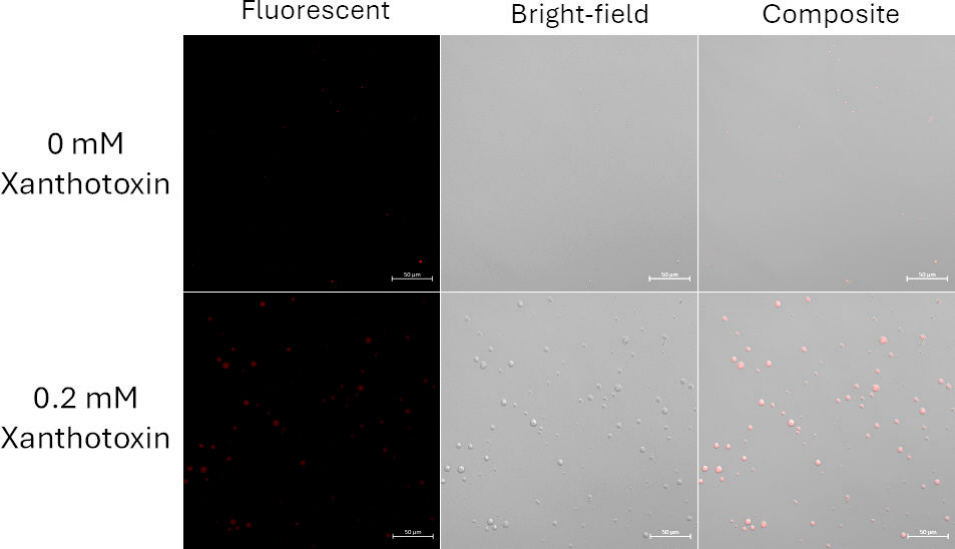
*Fusarium sporotrichioides* cultures in GYEP with or without 0.2 mM xanthotoxin. The supernatant was collected and stained with Nile red for visualization of lipid droplets.

Interestingly, trichodiene yields were not significantly different early in the fermentation despite large disparities in *F. sporotrichioides* biomass (day 4, 0.3 mM xanthotoxin; Fig. 3). This was likely due to the immense metabolic pressure induced by the xanthotoxin, as evidenced by the ratio of trichodiene to fungal biomass (Fig. 4). Fungal germlings were corpulent with lipid droplets, many of which floated free in the culture medium (Fig. S2). The presence of xanthotoxin did not significantly affect the morphology of the fungal germlings (Fig. S2). However, it did increase the number of free lipid droplets in the culture media (Fig. 5).

The size and the number of droplets increased considerably due to the addition of xanthotoxin and was visually apparent without the assistance of fluorescent dyes (Fig. S3). Oil skims readily formed in the small-scale fermentation vessels, becoming larger with increasing xanthotoxin concentration. The 0.2 mM xanthotoxin concentration was selected for pilot-scale fermentations to maximize trichodiene yield and minimize fungal growth inhibition.

### Pilot-scale fermentations of *F. sporotrichioides* F15

**Fig 6:**
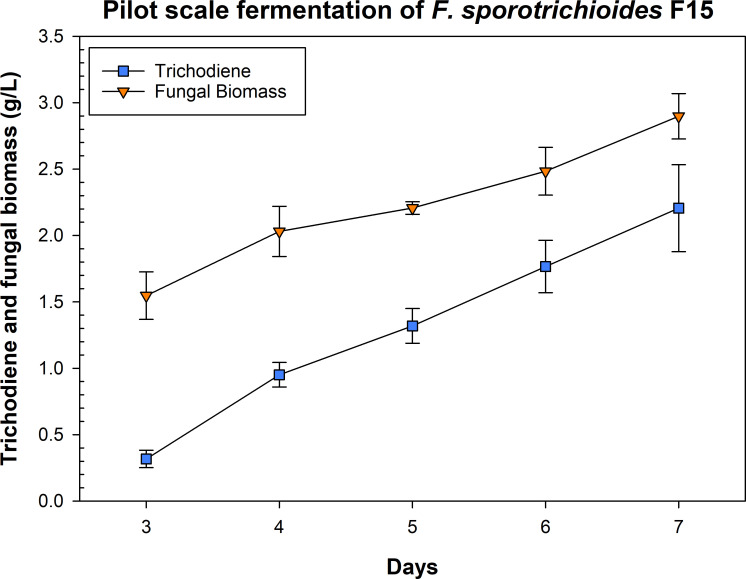
Trichodiene and fungal biomass yields from pilot-scale fermentations of *F. sporotrichioides* (F15). Error bars represent standard deviations.

Cultures of *F. sporotrichioides* grew vigorously in the pilot-scale fermentations and had a strong but pleasant odor of citrus and pine (author’s opinion) due to the accumulation of trichodiene. The culture pH was stable at 5.4–5.5 for the first 24 h but then steadily declined until 54 h, where it reached its nadir at a pH of 3.6–3.8. Afterward, the pH slowly increased to a range of 3.8–4.1, where it remained steady until the end of the fermentation. The pilot-scale fermentations had higher yields of trichodiene and greater trichodiene to fungal biomass ratios, 0.76 vs 0.66, compared to the flask fermentations at day 7 (Fig. 4). The day 7 yields of fungal biomass and trichodiene (in gram per liter, Fig. 6) were equivalent in the 30 L fermenters compared to the day 14 yields in the flask fermentations (0.2 mM, Fig. 3).

The 30 L cultures were collected and extracted with ethyl acetate, and the trichodiene was isolated using silica gel column separation. The purified trichodiene was evaluated via GC-MS before being used for contact and fumigation assays against the *F. graminearum* pathogen.

### Trichodiene inhibition of mycotoxin biosynthesis in *F. graminearum*

**Fig 7:**
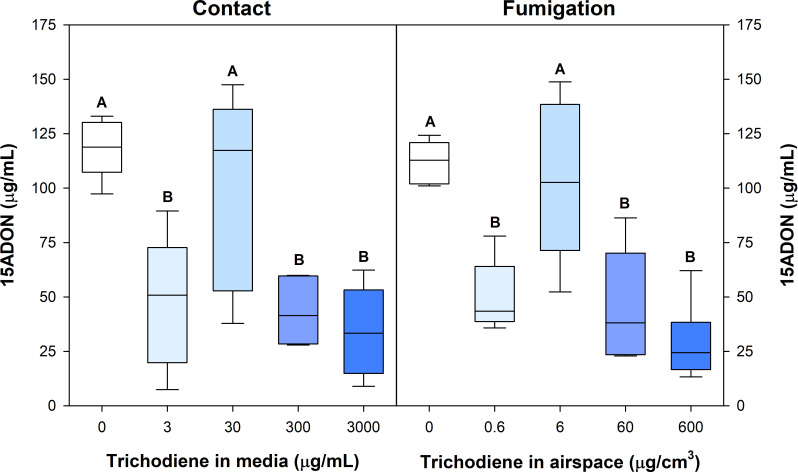
Contact and fumigation assays to assess the impact of trichodiene on 15ADON production by *F. graminearum*. Different letters represent statistically significant differences between trichodiene treatments at a given day, as determined by Student’s *t*-test; *α* = 0.05, *n* = 5; highest dose treatments were *n* = 10.

**Fig 8:**
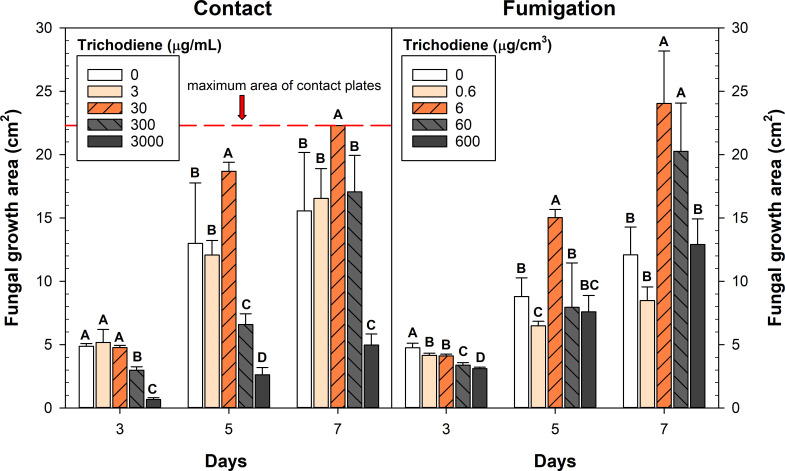
Impact of trichodiene treatments on fungal growth area. Error bars represent SE. Different letters represent statistically significant differences between trichodiene treatments at a given day, as determined by Student’s *t*-test; *α* = 0.05, *n* = 5; highest treatment dose in contact and fumigation assays was *n* = 10.

**Fig 9:**
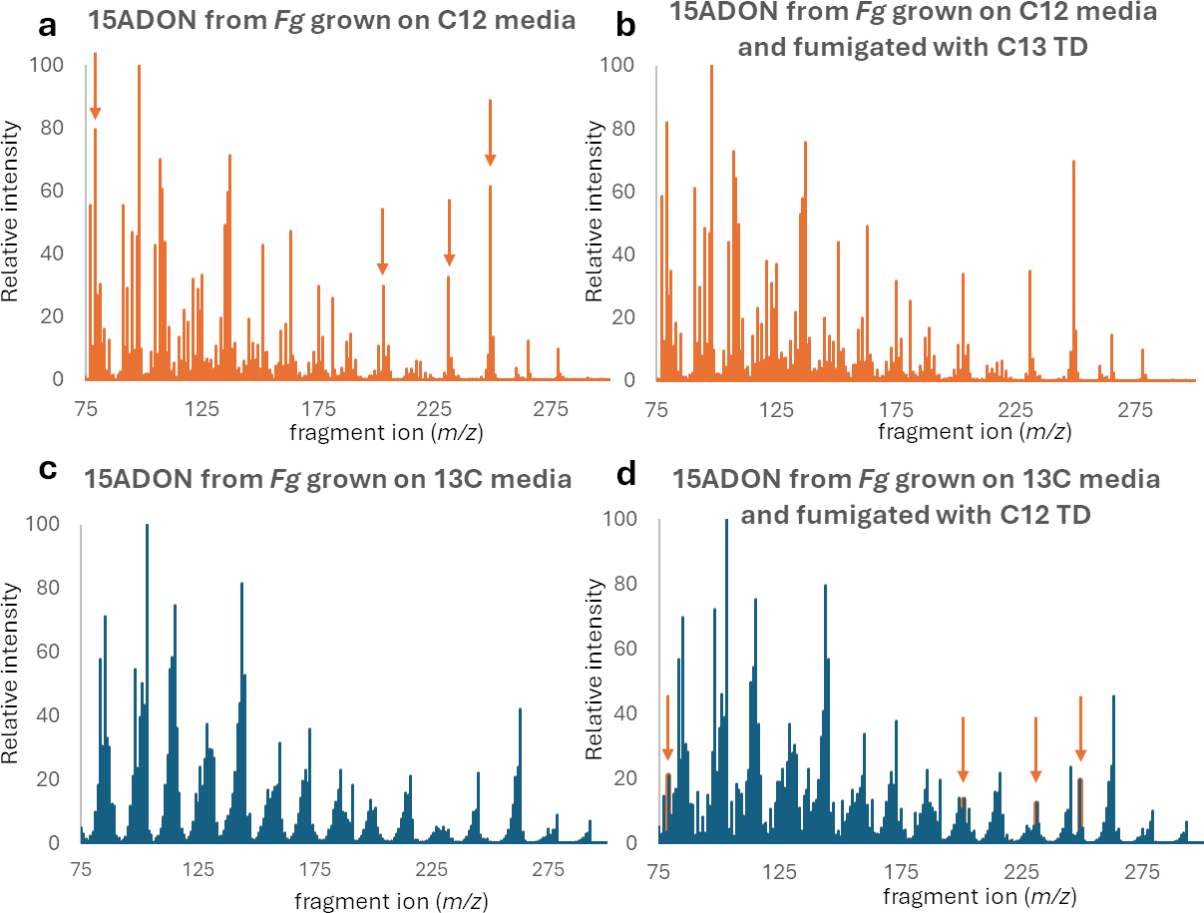
Gas chromatograms of 15ADON produced by *F. graminearum* (*Fg*). (**a**) Ion fragmentation pattern of ^12^C_17_-15ADON with characteristic peaks identified with orange arrows. (**b**) Ion fragmentation pattern of 15ADON from *Fg* grown on ^12^C_6_ glucose media that was fumigated by ^13^C_15_ trichodiene. (**c**) Ion fragmentation pattern of ^13^C_17_-15ADON. (**d**) Ion fragmentation pattern of 15ADON from *Fg* grown on ^13^C_6_ glucose media that was fumigated by ^12^C_15_ -trichodiene. Orange arrows identify characteristic ion fragments of ^12^C_17_-15ADON (82, 200, 231, and 249 *m/z*).

The trichodiene treatments had a concentration-dependent impact on mycotoxin production (Fig. 7). Trichodiene inhibited 15ADON production at all concentrations except 30 µg/mL and 6 µg/cm^3^, in both the contact and fumigation assays, respectively. This treatment concentration was highly variable but had statistically equivalent 15ADON accumulation compared with the control. The highest dose of trichodiene (3,000 µg/mL and 600 µg/cm^3^ for contact and fumigation, respectively) inhibited 15ADON accumulation by 71% in the contact and 74% in the fumigation assay. Interestingly, the lowest concentration of trichodiene tested for fumigation (3 µg/mL) was able to suppress mycotoxin biosynthesis and slow fungal growth in early development (Fig. 8 and 9; Fig. S4).

The trichodiene treatments also had a significant impact on fungal development, growth, and colony morphology (Fig. 8; Fig. S3 and S4). The highest doses of trichodiene in the contact assay (3,000 µg/mL) inhibited fungal radial growth by 68% after 7 days. Interestingly, the highest trichodiene dose (600 µg/cm^3^) for the fumigation treatments did not inhibit fungal radial growth after 7 days but did significantly inhibit 15ADON production (Fig. 8 and 9). This suggests that the suppression of mycotoxin biosynthesis was not primarily due to fungal growth inhibition. Unexpectedly, we observed that moderate- to high-dose treatments of trichodiene (30–3,000 µg/mL and 6–600 µg/cm^3^ for contact and fumigation, respectively) caused aerial hyphae formation in *F. graminearum*, a behavior we did not observe in the control. The 30 µg/mL and 6 µg/cm^3^ contact and fumigation treatments, respectively, caused a significant increase in fungal growth area compared to the control, though mycotoxin accumulation remained equivalent. The 60 µg/cm^3^ fumigation treatments also had a significant expansion of fungal growth area but accumulated 60% less 15ADON after 7 days (Fig. 8 and 9). This further suggests that the suppression of mycotoxin biosynthesis from externally applied trichodiene is not primarily associated with alterations in fungal growth. The effect of trichodiene fumigation was also evaluated on germinating wheat seeds; trichodiene fumigation had no effect on the ability or rigor of wheat germination and had no apparent impact on plant health (Fig. S6).

### Isotopically labeled trichodiene incorporation into trichothecene biosynthesis

As trichodiene is the fundamental backbone of all trichothecene mycotoxins, there was a concern that externally applied trichodiene could be incorporated into the toxin biosynthesis pathway and potentially increase toxin production. To investigate this possibility, we produced isotopically labeled ^13^C_15_-trichodiene from *F. sporotrichioides* F15 and ^13^C_17_-15ADON from the *F. graminearum* mutant strain B4-1 using ^13^C_6_ glucose as a sole carbon source. The B4-1 mutant was then either grown on ^12^C_6_ glucose media and fumigated with ^13^C_15_-trichodiene or grown on ^13^C_6_ glucose media and fumigated with ^12^C_15_-trichodiene. For the 15ADON from the B4-1 mutant grown on ^12^C_6_ glucose media and fumigated with ^13^C_15_-trichodiene, we did not observe ion fragments indicating ^13^C_15_-trichodiene incorporation into trichothecene biosynthesis (Fig. 9a and b). However, for the 15ADON from the B4-1 mutant grown on ^13^C_6_ glucose media and fumigated ^12^C_15_-trichodiene, we observed that a small proportion of 15ADON was synthesized from the volatile ^12^C_15_-trichodiene treatments (Fig. 9c and d). Examination of GC-MS data revealed the presence of ions (82, 200, 231, and 249 *m*/*z*) characteristic of ^12^C_17_-15ADON in cultures grown on ^13^C_6_ glucose-YEPD medium after fumigation with ^12^C_15_-trichodiene (Fig. 9d). This confirmed that while trichodiene fumigation can inhibit trichothecene production, minor amounts of trichodiene can also be incorporated into trichothecene biosynthesis.

## DISCUSSION

Fungal product utilization efforts can be stymied by difficulties in the large-scale production and isolation of promising compounds (32). Extensive research efforts must go into determining ideal growth conditions, the production of a high-yielding mutant strain, developing extraction methods, and finally optimizing scalability and costs. In this study, we demonstrated the pilot-scale fermentation and isolation of the non-phytotoxic fungal volatile trichodiene and its ability to mitigate mycotoxin contamination. The addition of xanthotoxin to the fungal media greatly increased trichodiene yield, but high concentrations were severely inhibitory to fungal growth, and a balance needed to be struck to maintain sufficient fungal biomass while maximizing yield. The production of trichodiene was dependent on two factors: (i) the use of a *F. sporotrichioides* mutant that accumulated trichodiene rather than T-2 toxin and (ii) the effect of the furocoumarin xanthotoxin on the release of trichodiene into the culture media. We selected the *F. sporotrichioides* (F15) mutant, rather than a previously used mutant strain of *T. harzianum* T34 (14), due to the ease of fermentation, rapid growth, high trichodiene yield, lack of significant foam production (a major issue in *Trichoderma*), and the subsequent ease of organic solvent extraction (as compared with *T. harzianum*, which can form substantial emulsion layers). Finally, it was also selected due to our previous observation that trichodiene production increased with xanthotoxin exposure (20). Xanthotoxin, otherwise known as 8-methoxypsoralen—a more palatable name for use in medical treatments—has been evaluated for anticancer, anti-inflammatory, antioxidant, and photosensitizing activity (33–36).

When added to the culture media, xanthotoxin significantly increased trichodiene accumulation and caused fungal lipid droplet release into the growth media (Fig. 5). This behavior was not observed in the cultures grown in xanthotoxin-free media. Xanthotoxin has been previously shown to selectively inhibit the fungal P450 enzymes Tri1 and Tri4, which are multifunction oxygenases involved in trichothecene production (20). This resulted in the accumulation of trichodiene in toxigenic *F. sporotrichioides*, as trichodiene could not be converted into isotrichotriol (26, 37). A number of furanocoumarins, aromatic plant secondary metabolites, have been observed to inhibit trichothecene biosynthesis (25, 38). Among the furanocoumarins tested, xanthotoxin caused the greatest accumulation of trichodiene, four- to eightfold greater than comparable treatments (25). However, this effect is not solely due to P450 inhibition, as xanthotoxin also increased trichodiene content in a *TRI4* knockout *F. sporotrichioides* strain (20). Furthermore, xanthotoxin treatment did not alter the expression of the trichodiene synthase encoded by *TRI5*.

It is still unclear how xanthotoxin causes trichodiene accumulation, but it may be due to changes in other regulatory pathways associated with fungal secondary metabolism, vesicles, and toxisome formation. In *F. graminearum*, once trichothecene biosynthesis is initiated, the endoplasmic reticulum (ER) undergoes significant reorganization to form ovoid structures called toxisomes that colocalize with key toxin-biosynthesis enzymes: Tri1, Tri4, and Hmr1 (39–41). At the same time in the ER, lipid droplet biogenesis is induced—both *in vitro* and in infected plant tissues—and a large number of lipid droplets accumulate in the fungal hyphae (39, 42). We observed this same effect in *F. sporotrichioides* in our liquid cultures, as the fungal germlings were filled with abundant lipid droplets (Fig. S2). Disruption of lipid droplet biogenesis, chemically or genetically, results in reduced pathogen virulence and mycotoxin production (42). When *F. sporotrichioides* was treated with xanthotoxin, we observed a substantial increase in the fungal export of lipid droplets into the growth media. This may have helped promote further lipid droplet production and subsequent export. Additional research is needed to fully elucidate this effect, and xanthotoxin may be a useful tool in studying mechanisms associated with trichothecene export/release during pathogenesis.

The regulation of trichothecene biosynthesis is highly complex. The pathogen employs specialized adaptations and intricate cellular compartmentalization for biosynthesis of the toxins (41, 43–45). Previous reports have shown that trichodiene, the volatile intermediate in trichothecene biosynthesis, also plays a role in modulating trichothecene biosynthesis (14, 39, 46). Interestingly, our results showed that externally applied trichodiene, either as a contact in media or fumigation treatment, disrupted trichothecene biosynthesis. In contrast, Seong et al. showed that the addition of trichodiene to liquid cultures of a *TRI5* mutant strain of *F. graminearum*, otherwise incapable of producing trichodiene, resulted in incorporation of trichodiene into trichothecene biosynthesis. This difference may be due to the very different environments and hypha growth in liquid media vs solid media (47).

In the current study, trichodiene inhibited trichothecene biosynthesis in a dosage-dependent, non-linear manner. Although the highest and lowest fumigation treatments of trichodiene were able to inhibit 15ADON accumulation by 74% and 55%, one of the intermediate treatment amounts resulted in no change in trichothecene production (Fig. 7). Even though we were able to demonstrate that some of the trichodiene biofumigant can be incorporated into 15ADON, none of the treatments resulted in an increase in 15ADON contamination. Nevertheless, further studies with multiple treatment amounts under variable conditions will need to be tested to eliminate this possibility. Utilization of trichodiene as a chemical control method to inhibit mycotoxin accumulation in crops will require careful formulation to ensure efficacy. However, previous results using the beneficial fungus *T. harzianum*, engineered to emit trichodiene, downregulated trichothecene biosynthesis and enhanced the wheat defense response, resulting in approximately 70% reduction in trichothecene accumulation in infected wheat (14).

The effects of secondary metabolites can often be concentration dependent. For example, the terpene (−)-α-pinene can modulate antimicrobial resistance in *Campylobacter jejuni* in a concentration-dependent manner through two separate modes of action (48). The volatile fungal polyketide 6-pentyl-α-pyrone (6-PP) from *Trichoderma*, a potent inhibitor of fungal growth, can either harm or enhance plant growth, depending on the concentration and application method (49–51). In seedling germination assays, even low doses of 6-PP caused substantial growth inhibition. However, for *Fusarium moniliforme* inoculated maize in greenhouse conditions, intermediate doses of 6-PP (200 mg/L) promoted plant shoot and root elongation (52). Our current results suggest that trichodiene may be useful at controlling mycotoxin contamination by trichothecene-producing pathogens alone or in combination with other antifungal treatments. Additional experiments are needed to assess trichodiene efficacy against other members of the *F. graminearum* species complex, which contains a wide diversity of fungi with specialized trichothecene production (53). Furthermore, trichodiene may be useful in combating trichothecene-producing members of the *Fusarium sambucinum* and *Fusarium incarnatum-equiseti* species complexes, which cause significant agricultural damage in potatoes and legumes (54). With the development of our pilot-scale fermentation method to produce trichodiene, these investigations are now possible.

Trichodiene may be especially useful for formulations designed for field applications as we observed no phytotoxic effect on wheat, consistent with previous reports on *Arabidopsis* (15). Trichodiene has been found to induce plant defense-related gene expression, mainly those belonging to the salicylic acid pathway (55, 56). The plant defense induction can be so strong as to reduce aboveground plant growth and lateral root development in tomatoes (55). However, we did not observe a reduction in wheat germination success or seedling germination vigor in our fumigation treatment range. Further method optimizations are necessary to convert pilot-scale production to the industrial scale yields necessary for commercial field applications. The use of volatile compounds, such as trichodiene, for pathogen control can be challenging in field settings due to application and volatilization rates. We are currently investigating trichodiene as a fumigation treatment in controlled environments, such as during barley/wheat malting, where mycotoxin contamination poses significant food safety concerns.

This research describes the pilot-scale production, isolation, and demonstrated impact of *F. sporotrichioides*-derived trichodiene to suppress mycotoxin accumulation in the *F. graminearum* pathogen. We determined that xanthotoxin is necessary to induce significant accumulation of trichodiene in the fungal ferment and that it is likely involved in lipid droplet export from fungal tissue. The impact of fermentation time, extraction solvent, and filtration on the overall trichodiene yield was also described. Based on these research findings, trichodiene can be produced using common large-scale fermentation methods. We can now investigate field formulations that utilize its novel mechanism of control to suppress mycotoxin contamination in food and feed.

## Data Availability

The data sets generated during the current study are available from the corresponding author upon reasonable request.

## SUPPLEMENTAL MATERIAL

Supplemental material aem.01695-25-s0001.docx Fig. S1 to S6; Table S1.

ASM does not own the copyrights to Supplemental Material that may be linked to, or accessed through, an article. The authors have granted ASM a non-exclusive, world-wide license to publish the Supplemental Material files. Please contact the corresponding author directly for reuse.
