# Supplementary material for: Pilot-scale fermentation to produce trichodiene, a fungal volatile that suppresses trichothecene production in mycotoxigenic fungi
Source: Appl Environ Microbiol. 2026 Jan 15;92(2):e01695-25. doi: 10.1128/aem.01695-25 (PMC12915363; doi:10.1128/aem.01695-25)
Supplement: Supplemental material — Fig. S1 to S6; Table S1. [file aem.01695-25-s0001.docx]

**Supplemental Information**:


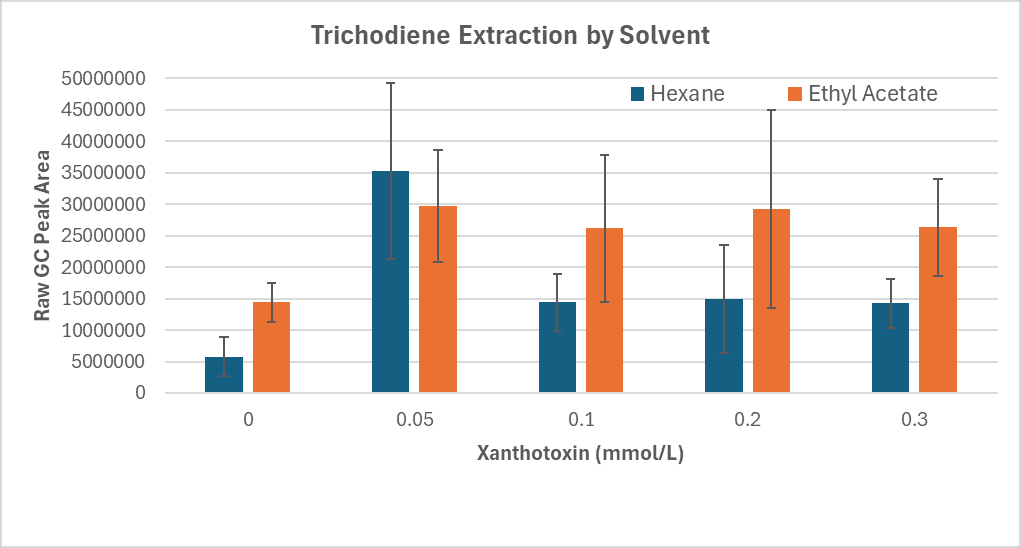


**Supplemental Figure 1**: Relative trichodiene yield from hexane or ethyl acetate organic solvent extractions of *F.* *sporotrichioides* grown with varying concentrations of xanthotoxin. Error bars represent SD; n=3.


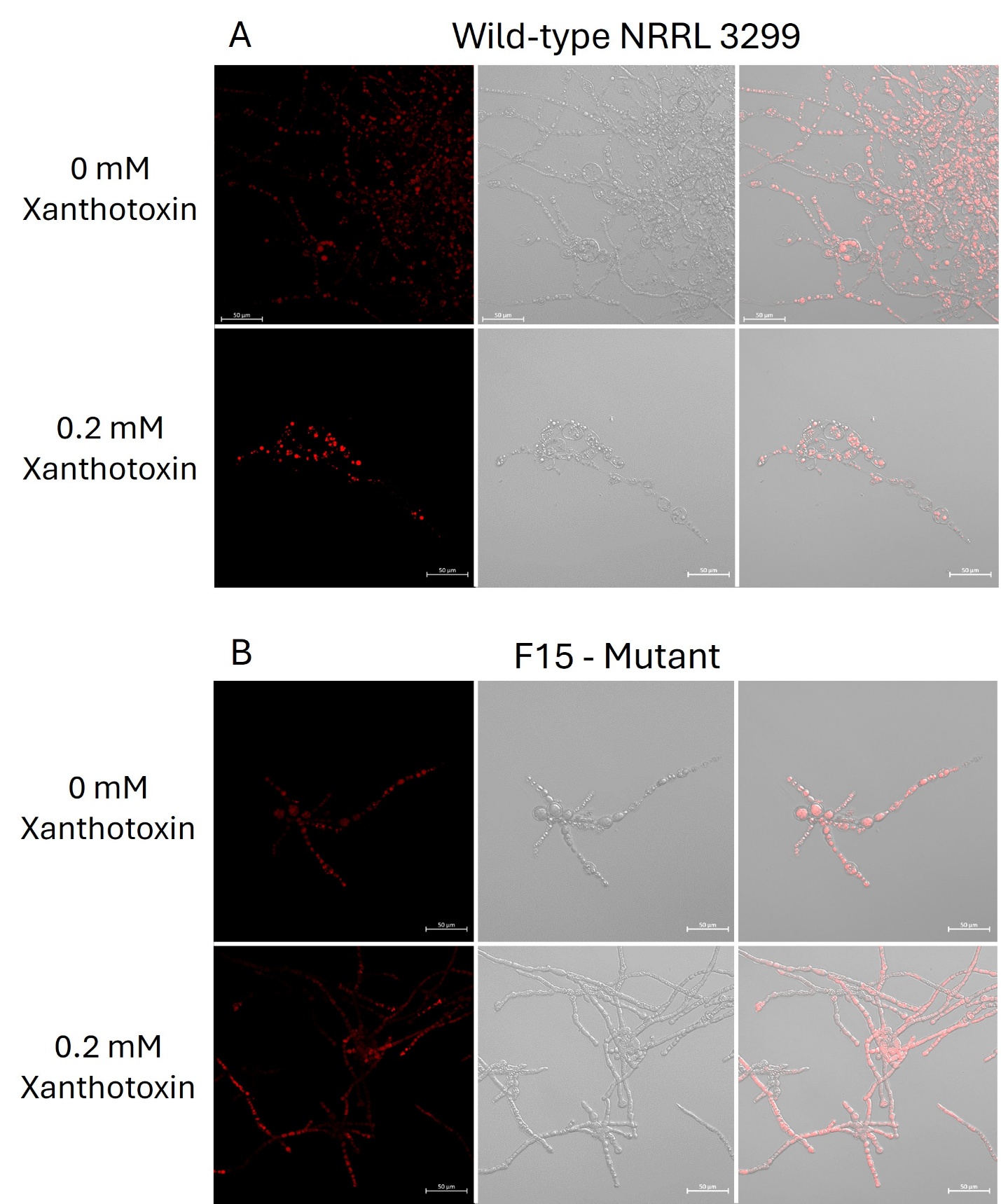


**Supplemental Figure 2**: Image of *Fusarium sporotrichioides* Wild-type NRRL 3299 and the mutant F15 strain grown with and without 0.2 mM xanthotoxin.


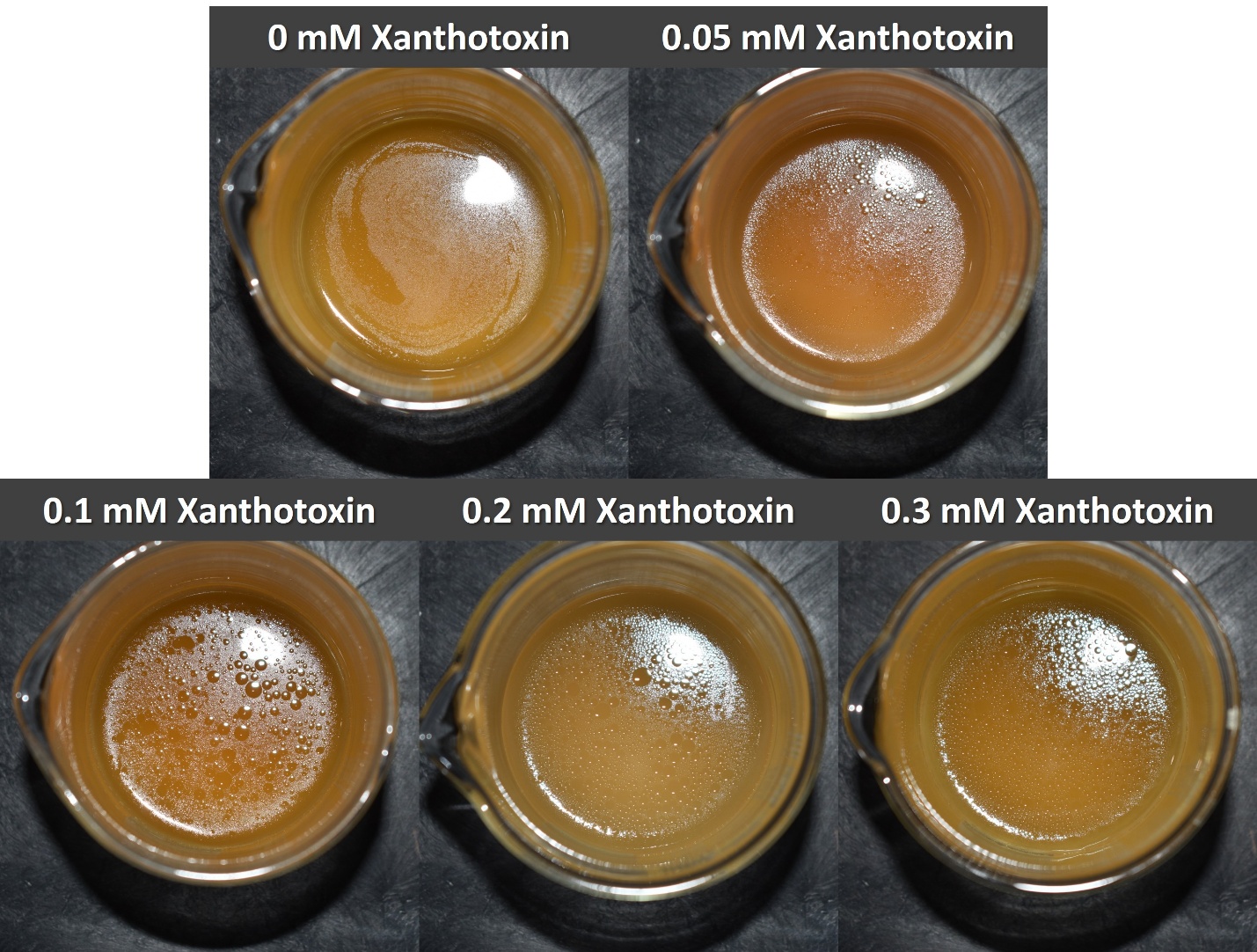


**Supplemental Figure 3**: Representative images of *Fusarium sporotrichioides* (F15 strain) grown with 0, 0.05, 0.1 0.2, 0.3 mM xanthotoxin concentration. Oil droplets are apparent on the surface of the culture media (9 days post spore inoculation).

| Repetition | Biomass of 20mL Spore-grown Inoculum (mg) | Biomass of 20mL Plug-grown Inoculum (mg) |
| --- | --- | --- |
| 1 | 93.9 | 57.7 |
| 2 | 92.4 | 51.2 |
| 3 | 86.5 | 52.6 |
| Average | 90.93^A^ ±3.91 | 53.83^B^ ±3.42 |

**Supplemental Table 1**. Fungal biomass from spore-grown and plug-grown inoculum. Cultures were 5 day old 100 mL cultures of *Fusarium sporotrichioides* F15. The cultures were filtered and subsequently lyophilized for biomass determination.


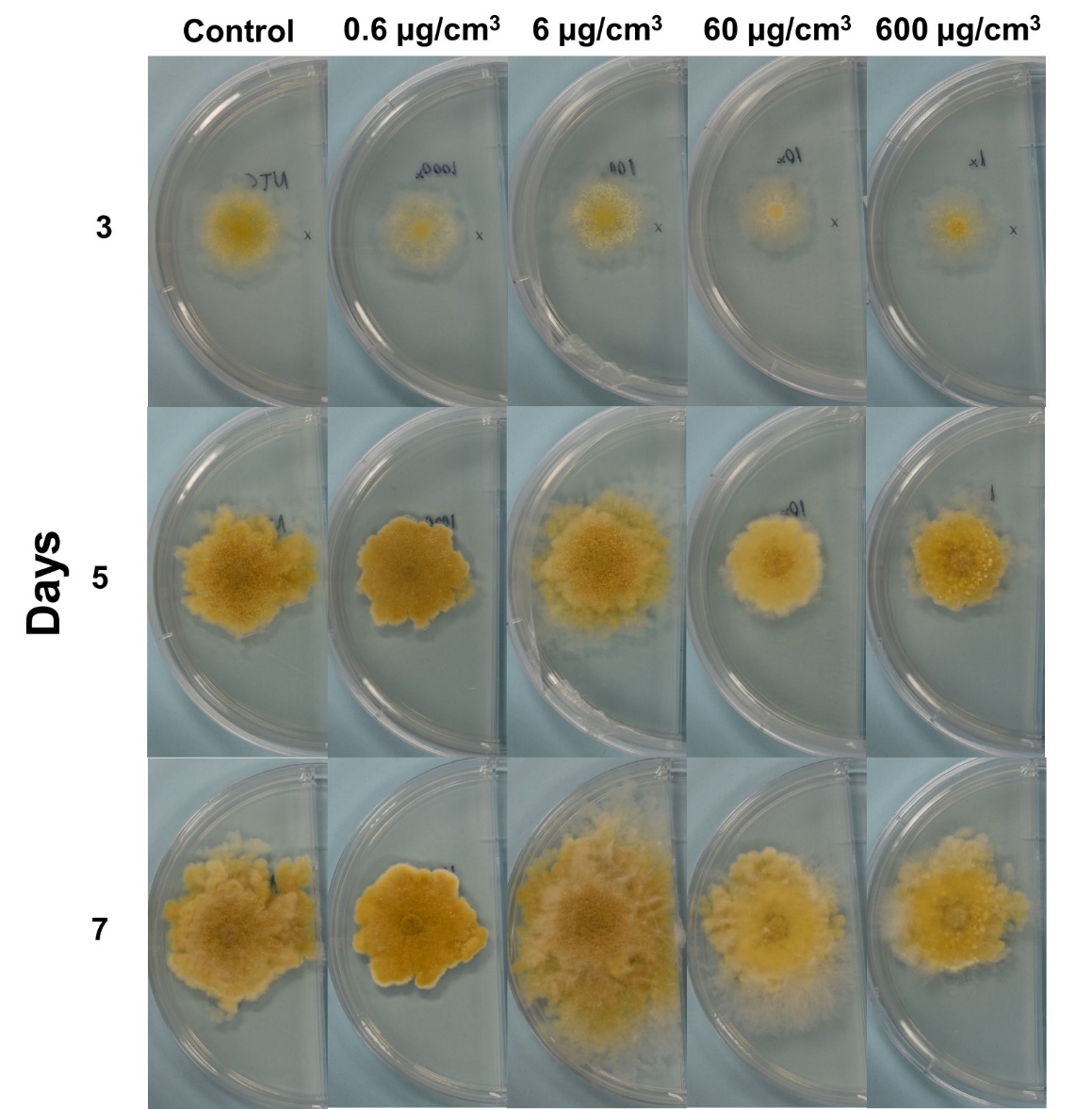


**Supplemental Figure 4**. Impact of trichodiene fumigation on *Fusarium graminearum* (Gz3639) fungal growth area.

**
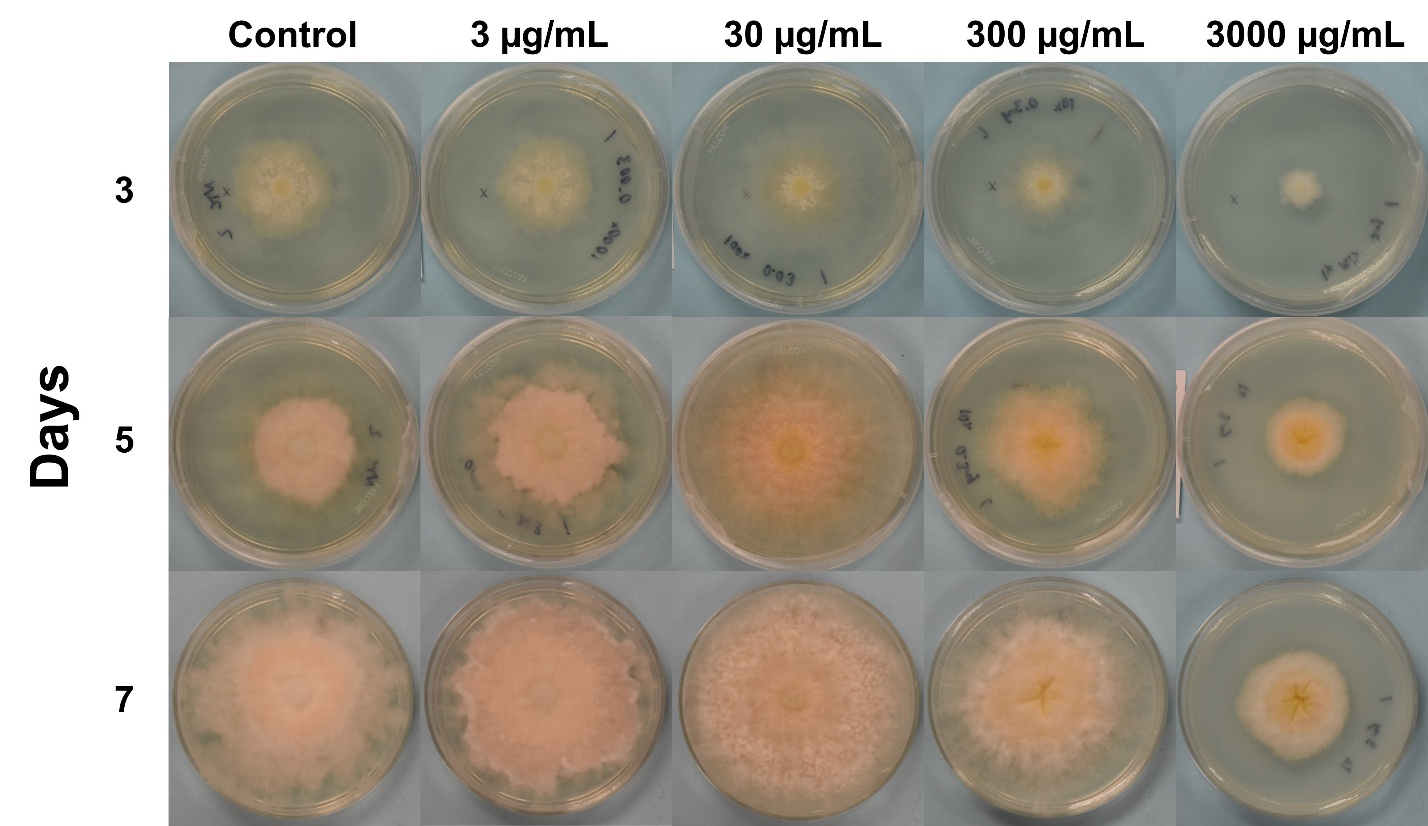
**

**Supplemental Figure 5**. Impact of trichodiene on *Fusarium graminearum* (Gz3639) fungal growth area. Contact assays.


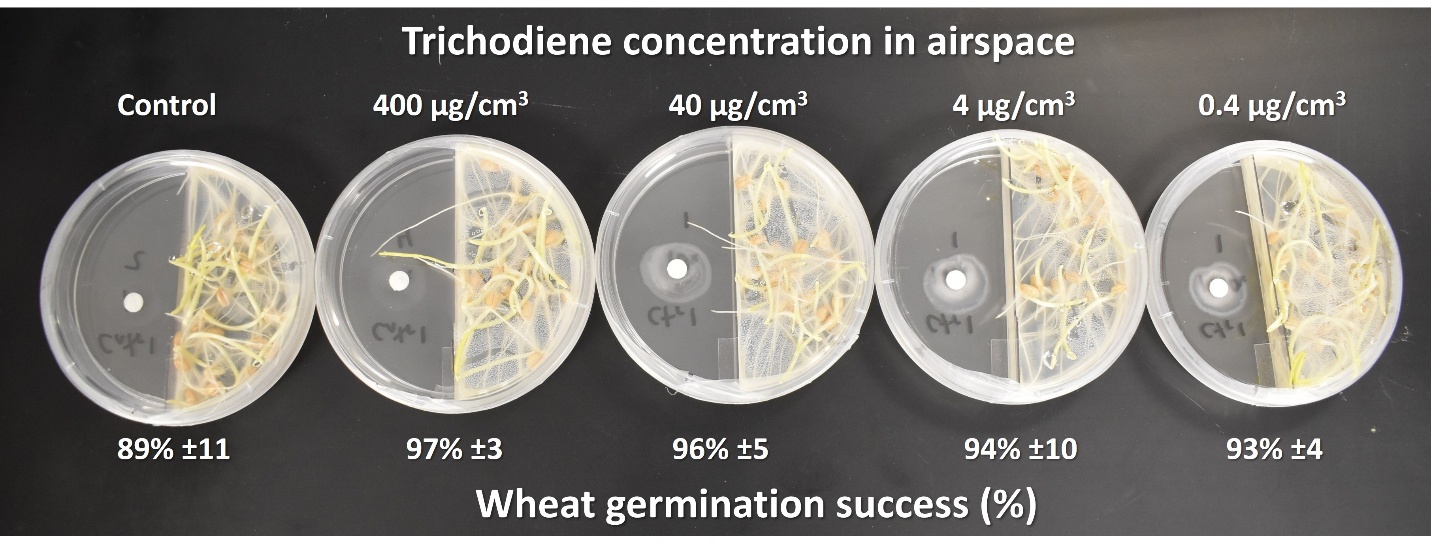


**Supplemental Figure** **6**. Wheat germination success when treated with various concentrations of trichodiene fumigant. Twenty wheat seeds were allowed to germinate for 5 days under continuous trichodiene fumigation (n = 5).
